# Coefficient of Variation to Assess the Reproducibility of Meal-Induced Glycemic Responses: Development of a Clustering Algorithm

**DOI:** 10.2196/68821

**Published:** 2025-11-20

**Authors:** Nicole Lubasinski, Hood Thabit, Paul W Nutter, David Petrescu, Simon Harper

**Affiliations:** 1 Department of Computer Science, The University of Manchester, Kilburn Building, Oxford Road, Manchester, M13 9PL, United Kingdom, +44(0)1613069280; 2 Diabetes, Endocrine and Metabolism Centre, Manchester Royal Infirmary, Manchester, United Kingdom; 3 Division of Diabetes, Endocrinology and Gastroenterology, School of Medical Science, The University of Manchester, Manchester, United Kingdom

**Keywords:** bolus targeting solution, meal-based PPGR clustering algorithm, postprandial glycemic response (PPGRs), PPGR clusters, reproducibility, type 1 diabetes (T1D)

## Abstract

**Background:**

Managing type 1 diabetes (T1D) requires maintaining target blood glucose levels through precise diet and insulin dosing. Predicting postprandial glycemic responses (PPGRs) based solely on carbohydrate content is limited by factors such as meal composition, individual physiology, and lifestyle. Continuous glucose monitors provide insights into these responses, revealing significant individual variability. The statistical clustering method proposed here balances the number of clusters formed and the glycemic variability of the PPGRs within each cluster to offer a clustering technique on which treatment decisions could be based.

**Objective:**

This study aims to develop and evaluate a PPGR clustering method that identifies reproducible meal-specific glucose patterns in people with type 1 diabetes.

**Methods:**

Blood glucose data from the OhioT1DM dataset were used to assess clustering of PPGR based on the coefficient of variability (CV) of glucose. Clustering was performed using statistical clustering, with each PPGR isolated into 48 data points per event. A CV threshold of <36% was used to define clinically similar clusters. This aimed to cluster PPGRs with minimal glycemic variability. The approach aims to enhance precision in analyzing postprandial glycemic dynamics, assessing cluster cohesion via standard deviation and CV within meal categories.

**Results:**

The analysis revealed a reproducible set of PPGR clusters specific to meal types and individuals (mean [SD], 2.4 [1.8] for breakfast, 2.7 [0.9] for lunch, and 3.1 [1.0] for dinner), with the number of clusters varying across participants and meals in the dataset. Carbohydrate intake alone did not affect cluster formation, suggesting a complex relationship between meal composition and PPGR variability. However, certain individuals showed significant associations between carbohydrate intake and cluster formation for specific meals.

**Conclusions:**

The meal-based glycemic clustering algorithm provides a promising framework for predicting PPGRs in people with type 1 diabetes, independent of carbohydrate intake. It emphasizes the need for personalized prediction models to optimize time in range and enhance diabetes management. Efforts to refine treatment strategies are crucial in reducing T1D-related complications.

## Introduction

Type 1 diabetes (T1D) is a chronic autoimmune disease that inhibits the production of insulin, preventing the intrinsic regulation of blood glucose (BG) [1]. The management of T1D focuses on the international consensus of achieving BG levels within the recommended target range of between 3.9 and 10 mmol/L at least 70% of the time [2], while consuming a balanced and varied diet [3]. The key to optimal insulin therapy is minimizing BG fluctuations after meals [4] (postprandial glycemic response [PPGR]) and keeping the BG within the target range [2,5]. PPGR management in people with T1D (PwT1D) is further complicated by the need to administer the appropriate dose of exogenous bolus insulin [6], which is commonly determined by the amount of carbohydrate present in the meal [7]. Current treatment strategies focus on carbohydrate-based insulin dosing where people with T1D are required to determine the amount of carbohydrates present in the meal to calculate the appropriate bolus insulin dose needed [8]. The intricate interplay of individual physiology, metabolism, behaviors, and the diverse nature of meals in the real world, which impact the PPGR, complicates effective insulin therapy management [9]. Despite efforts to refine carbohydrate-based insulin dosing strategies, factors beyond carbohydrate content significantly influence the PPGR, including fiber content, exercise, and metabolic factors [10-12], revealing a nuanced and complex landscape [3,13-16]. However, to optimize the PPGR and effectively keep BG levels in range, the bolus dosage and timing should factor in the speed at which the carbohydrates are absorbed, which is determined by other nutritional factors, such as amount of fat, protein, and fiber [17].

The use of continuous glucose monitors (CGMs) allows for a complete picture of the PPGR to a meal to be seen and analyzed [18]. It has been shown that PPGRs are reproducible within individuals (intraindividual) when exposed to meals of the same nutritional composition [5,15,19]. However, interindividual variability in PPGRs to identical meals has been noted in the literature [20,21], suggesting that individual characteristics influence the PPGR beyond what is expected from the meal itself [17,22]. However, this intraindividual reproducibility offers the potential for an effective PPGR self-management technique in T1D based on the learned response to the previous PPGR to a given meal [23]. Leveraging this repetitiveness of PPGRs, the ability to isolate PPGR clusters will allow for meal-based glycemic responses that exhibit similar patterns to be effectively grouped together. Being able to understand and predict how one might respond to a meal based on its composition, the physiology of the individual, and the lifestyle factors that influence BG levels allows for reduction in the burden of self-management and the potential to optimize meal and bolus planning [14,22,24].

Clustering PPGR data can reveal relationships between variables and uncover patterns in glycemic responses to meals, enabling tailored treatment interventions based on individualized PPGR profiles [14]. In this work, a statistical clustering approach was developed to balance the number of clusters with the glycemic variability within each cluster, using variability as a measure of internal cohesion. The coefficient of variability (CV) of glucose, which quantifies glycemic variability over time [25], was adopted as the primary parameter for assigning PPGR events to specific clusters. By constraining the CV within each cluster to below 36%, the method aims to identify repeated PPGR patterns for specific meals, potentially reducing hypoglycemia risk when applied to guide insulin therapy [25]. The balance between cluster quantity and clinically relevant measures of glycemic variability is particularly important given the individualized nature of PPGR responses, where the optimal number of clusters may differ between subjects. In evaluating various clustering strategies, conventional methods, including Time Series Means Clustering [26,27], standard partitioning methods [28,29], DBScan [30], and hierarchical clustering [31], proved unsuitable due to limitations in handling small datasets, irregular data densities, and the individualized nature of glucose response patterns. In contrast, the proposed CV-based clustering method is designed to overcome these limitations, providing a framework for analyzing personalized glucose response dynamics.

This work focuses on identifying the repetitive patterns of PPGRs elicited within specified meal categories (ie, breakfast, lunch, and dinner) and generates clinically similar PPGR clusters for people with T1D, which minimize the glycemic variability and have a low risk of hypoglycemic outcomes [25,32]. This will provide a categorization system that captures the reproducibility of everyday PPGR events, allowing for a personalized and adaptive BG prediction model that is based on unique PPGR events. This could be extrapolated to specific meals offering the ability to learn the appropriate meal-time insulin bolus (bolus targeting solution) to optimize the time in range (TIR).

The overarching aim is to utilize the PPGR clustering technique to provide a practical strategy for insulin therapy treatment adjustments, ultimately improving TIR for people with T1D.

## Methods

### Patient Data

This study evaluated whether PPGRs could be matched and clustered using the CV of the PPGR. Data were from the OhioT1DM dataset [23], comprising 8 weeks of CGM and self-reported lifestyle logs from 12 adults with T1D using insulin pump therapy and Medtronic Enlite CGM systems. CGM readings were taken every 5 minutes, alongside insulin dosages, heart rate, and timestamped dietary and lifestyle events. Data were aligned to a 24-hour clock and stratified by day and week. Despite its modest size [33], the dataset reflects real-world conditions and is widely used in T1D research [34]. Participant demographics are shown in Table 1.

**Table 1.** Gender, age range, insulin pump, and insulin used along with the continuous glucose monitors (CGM) for each OhioT1DM participant as available from the dataset [23].

ID	Gender	Age range (years)	CGM device	Pump and insulin used
540	Male	20‐40	Medtronic Enlite	630G (Humalog)
544	Male	40‐60	Medtronic Enlite	530G (Humalog)
552	Male	20‐40	Medtronic Enlite	630G (Humalog)
567	Female	20‐40	Medtronic Enlite	630G (Humalog)
584	Male	40‐60	Medtronic Enlite	530G (Novolog)
596	Male	60‐80	Medtronic Enlite	530G (Humalog)
559	Female	40‐60	Medtronic Enlite	530G (Novolog)
563	Male	40‐60	Medtronic Enlite	530G (Humalog 200)
570	Male	40‐60	Medtronic Enlite	530G (Humalog)
575	Female	40‐60	Medtronic Enlite	530G (Novolog)
588	Female	40‐60	Medtronic Enlite	530G (Novolog)
591	Female	40‐60	Medtronic Enlite	530G (Novolog)

### Ethical Considerations

This study is an in silico analysis that relies exclusively on open-access, publicly available data obtained from recognized repositories. No new data were collected, and no interaction or intervention with human participants, animals, or identifiable private information occurred. As such, this research does not meet the criteria for “human subjects research” as defined by institutional and international ethical standards. Ethics approval was not required for this study as it utilized open-access, deidentified data available in the public domain. The analysis involved no direct or indirect contact with human participants or animals, and no identifiable personal data were used [35-39].

### Isolating PPGR Process

**Figure 1. F1:**
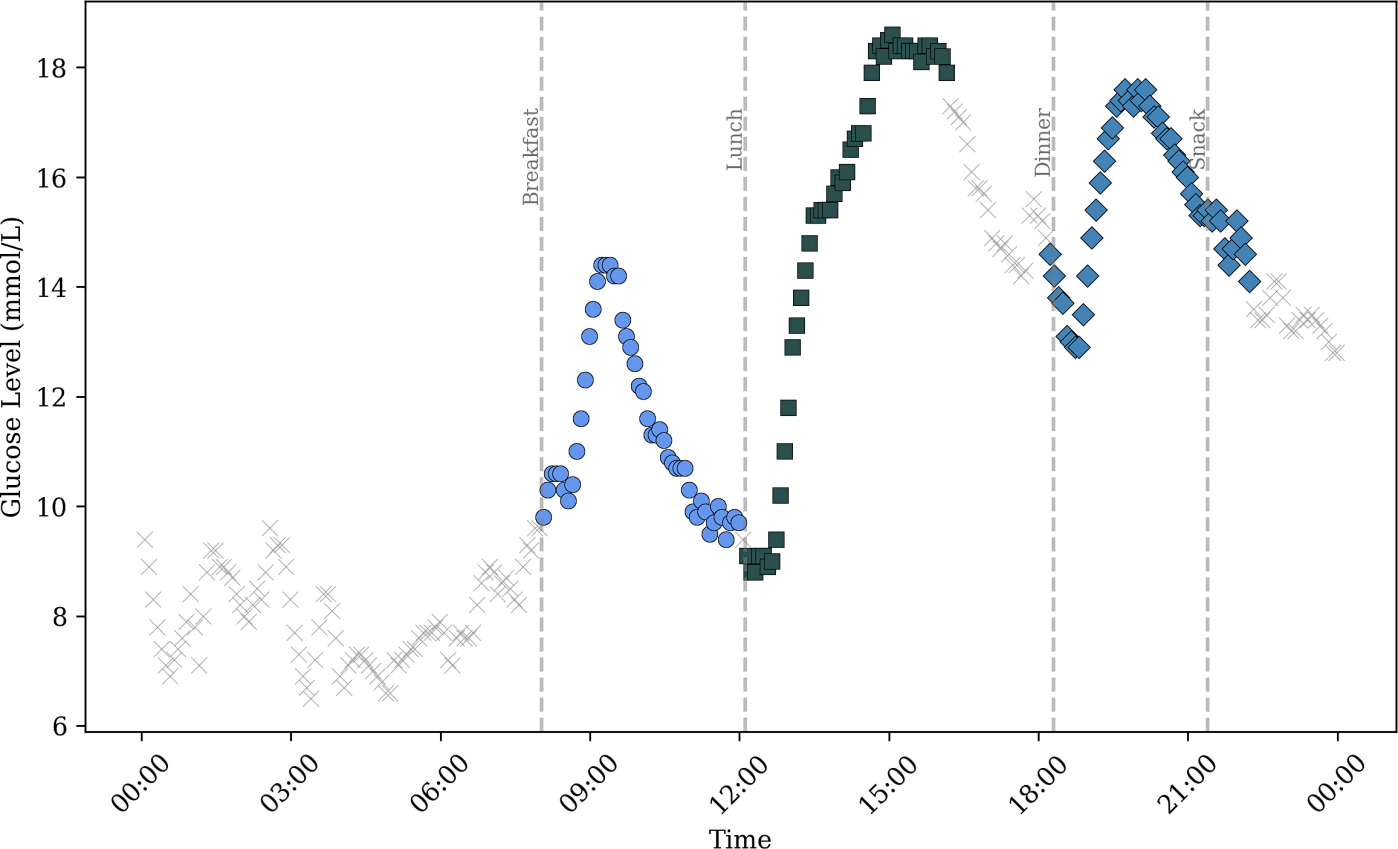
Example of mealtime postprandial glycemic response (PPGR) isolation from a single day of blood glucose data from a single participant from the OhioT1DM dataset (544). The different colors and markers represent the distinct meal categories, “x” indicating unassigned BG data, “🔵 ” indicating breakfast PPGR, “⬛️ ” the lunch PPGR and “🔷 ” dinner PPGR. The dashed gray lines indicate the time of the self-reported meal consumption.

A PPGR was defined as a 4-hour window (48 readings) from self-reported mealtime or bolus insulin administration (see Figure 1). When mealtimes were missing, start times were imputed from matching events on the same weekday in other weeks, using the closest average timing. The 4-hour window was chosen to capture the full glycemic excursion, given that glucose in nondiabetics typically normalizes within 90 minutes [40], gastric emptying completes within 4‐5 hours [41], and rapid-acting insulin (eg, Novorapid) peaks at 100‐120 minutes when given 15‐20 minutes before meals [42]. Events with <2 hours of CGM data (24 points) were excluded.

PPGRs were categorized as breakfast (06:00-10:00), lunch (10:00-14:00), snack (14:00-18:00), or dinner (18:00-22:00) in line with circadian and sociocultural eating patterns [41,43,44]. Additional carbohydrate intake within the 4-hour postprandial window was considered part of the original PPGR. A bolus is classified as meal-related if its start time falls within a tight window around a logged meal, specifically from 4 minutes before to 4 hours after the meal; boluses outside every such window are labeled corrections. Correction boluses are then summarized by the PPGR categorization bands (6-10, 10-14, 14-18, 18-22). Where bands did not have an isolated PPGR from a self-reported meal of mealtime bolus, the PPGR was isolated, and where the bands contained meal events, the bolus dose was confirmed to be a correction.

### Normalization of PPGR Events

To enable cross-comparison of glucose responses independent of absolute magnitude, each PPGR was normalized by centering it around its peak value. Specifically, the peak glucose reading within each 4-hour window was identified, and all other values were recalculated by subtracting this peak value. This process generated a normalized, peak-centered time series for each event, allowing for uniform trend analysis across individuals and events.

### Calculation of CV

The CV quantified variability by dividing the standard deviation of each isolated PPGR by the mean of all PPGRs for the same individual and meal category, then multiplying by 100 (Equation 1). This approach provided a normalized measure of glycemic variability contextualized within each person’s own physiological response patterns. A CV threshold of less than 36% was adopted as the criterion for acceptable variability, consistent with clinical literature associating lower CV values with reduced risk of hypoglycemia [25,45]. Given that one would expect the PPGR-specific CV to be significantly higher than the 24-hour CV, using the 24-hour threshold of 36% would ensure the internal cohesion of the PPGRs is tighter than the clinically recommended glycemic variability.


(1) $$CVPPGR=\left(\frac{\sigma_{\text{PPGR}}}{\mu_{\text{PPGR}}}\right)\times 100$$

- 𝜎PPGR is the standard deviation of the isolated PPGR.
- *μ*PPGR is the mean of all the PPGRs in the same meal category for that individual.

### Clustering of Postprandial Glycemic Responses

**Figure 2.**
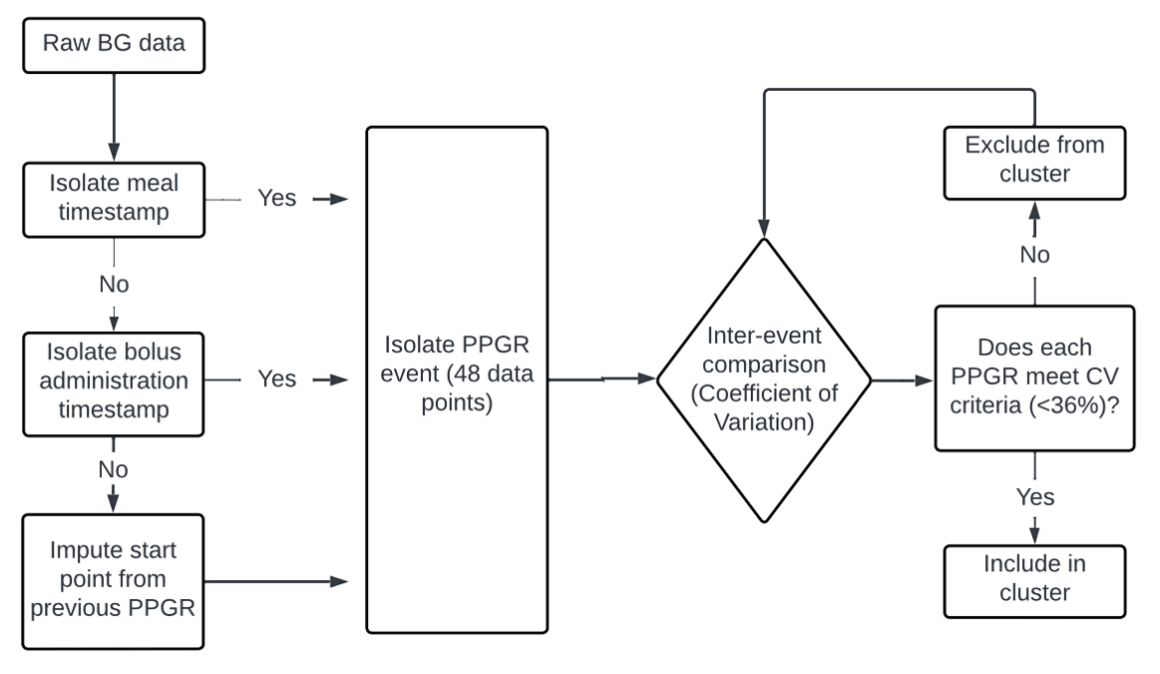
Methodological framework for assessing the reproducibility of postprandial glycemic responses (PPGRs): an iterative coefficient of variability (CV)-based clustering approach. BG: blood glucose.

Each PPGR was treated as a unique time series and clustered within the specified meal categories. An iterative pairwise algorithm began with an unassigned PPGR and added other unassigned events if the cluster CV remained <36%. Clusters were expanded until no additional events could be included without exceeding the threshold. Unassigned events were reevaluated in later iterations; PPGRs eligible for multiple clusters were assigned to the largest cluster. Events failing all inclusion criteria were labeled outliers (Figure 2).

### Cluster Evaluation

Each cluster was evaluated using several statistical measures to assess internal consistency and clinical relevance. The standard deviation of glucose values within each cluster was used to estimate dispersion around the cluster mean. The standard error was calculated by dividing the standard deviation by the square root of the number of PPGR events in the cluster, providing a measure of the reliability of the cluster mean. An internal CV for each cluster was also computed, based on the combined glucose values of the cluster and the overall mean for the corresponding meal category. The total number of PPGRs per cluster was recorded to assess distribution across clusters and ensure adequate representation.

### Statistical Analyses

Sample size and power calculations used the TTestIndPower function from statsmodels in Python [46], targeting power=0.8 and *α*=.05 to minimize type I or II errors [47]. Within each meal category, one-way ANOVA tested for differences in mean carbohydrate intake between clusters.

### Tools and Software

Data preprocessing, normalization, and clustering were implemented in Python 3.10.7 [48] using Visual Studio Code 1.77.3 [49] and standard libraries. The 36% CV threshold, though derived from 24-hour glucose variability, was used to ensure PPGR clusters had tighter internal cohesion than recommended clinical variability limits.

## Results

**Figure 3.**
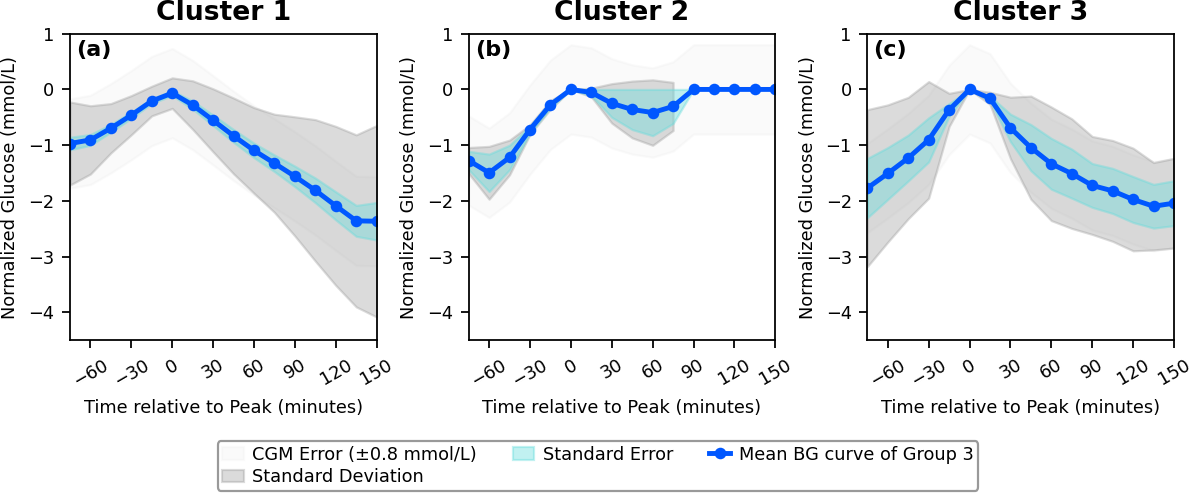
A sample of postprandial glycemic response (PPGR) clusters formed for a single participant. The plots reflect the mean blood glucose (BG) value of each formed cluster with the meal category breakfast, with indication of the standard deviation, error of the clustering technique, and the inherent error of the blood glucose sensor.

The 12 participants in the OhioT1DM dataset generated 20635 PPGRs, which reflects the consolidation of 99121685 blood glucose data points. The meal-matched isolated PPGRs were subjected to the CV-based clustering technique outlined above. It was found that the available BG data formed a set number of PPGR clusters for each meal type, with breakfast having mean (SD) of 2.4 (1.8) clusters identified, lunch having 2.7 (0.9) clusters, and dinner having 3.1 (1.0) clusters. An example of the clusters formed across all meals for a single participant is represented in Figures3^5^ [Fig F4][Fig F1]. This outlines the mean curve, standard deviation, and standard error of the cluster, in comparison to the standard error of the CGM device. The number of PPGR clusters present for each participant across the 3 meals, along with metrics and CV score, is outlined in Table 2, suggesting the reproducibility of PPGR events is specific to the individual and type of meal consumed. In the meal categories where the average CV is above 36%, this is due to the formation of outlier clusters, which contain individual PPGRs due to the PPGR-specific large CV. The cluster-specific metrics can be found in Table S1 in Multimedia Appendix 1. Along with the set number of PPGR clusters per meal type within each participant, there is variation in carbohydrate intake across meal type and individuals (Table 2). The individual cluster error across all clusters and all participants falls below the standard CGM error (0.8 mmol/L) [50] in all but 1 of the clusters formed, which was composed of outliers (563 lunch). The metrics for each isolated PPGR cluster, including the number of PPGRs isolated to each cluster, can be found in Table S1 (Multimedia Appendix 1).

When assessed on an individual basis, the distribution of carbohydrate intake was the same across all clusters in all meal categories barring 2 meals across 2 participants. For dinner in participant 563, the carbohydrates consumed had a significant effect on the cluster formation (*F*(2,42) = 3.48, *P*=.04) and lunch for participant 559 *F* (2,59)=4.66, *P*=.01. With the OhioT1DM sample size of 12 participants, the analysis revealed an effect size (Cohen *d*) [51] of 1.1968. The effect size of 1.1968 observed in this study suggests that the intervention had a strong impact [52].

**Figure 4. F4:**
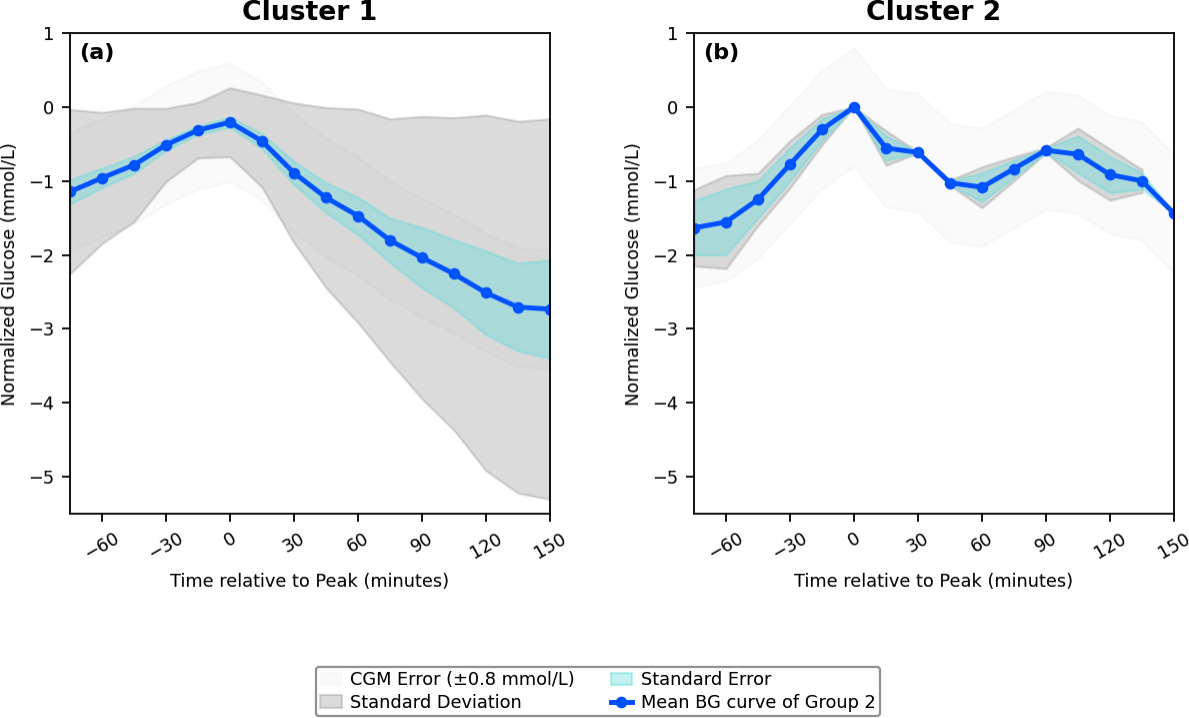
A sample of postprandial glycemic response (PPGR) clusters formed for a single participant. The plots reflect the mean blood glucose (BG) value of each formed cluster within the meal category lunch, with indication of the standard deviation, error of the clustering technique, and the inherent error of the blood glucose sensor.

**Figure 5.**
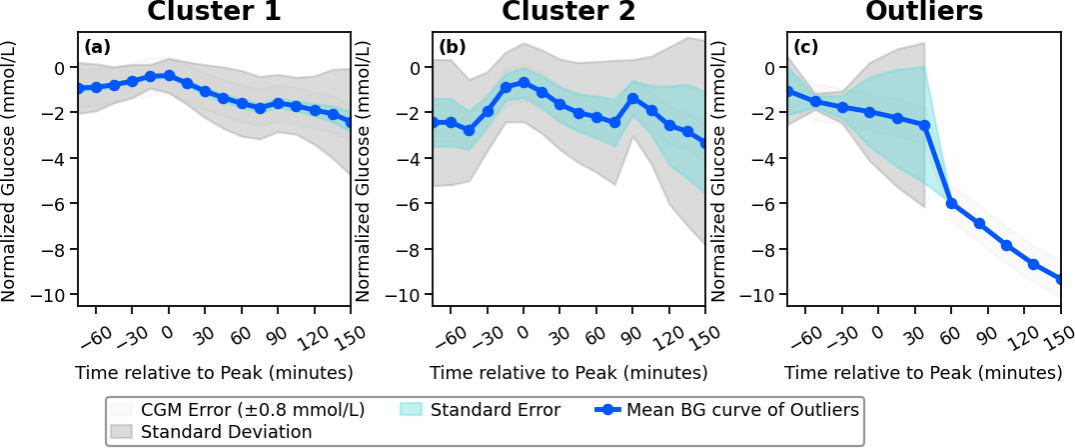
A sample of postprandial glycemic response (PPGR) cluster formed for a single participant. The plots reflect the mean blood glucose (BG) value of each formed cluster within the meal category dinner, including outliers (c), with indication of the standard deviation, error of the clustering technique, and the inherent error of the blood glucose sensor.

**Table 2.** The average number of clusters formed for each meal category, along with the within-cluster metrics (standard deviation, standard error, and coefficient of variability [CV])^a^.

ID	Meal	Clusters formed					Outliers				Statistical values	
		Number of clusters formed	PPGRs^b^ per cluster, mean (SD)	Peak BG^c^ (mmol/L), mean (SD)	Internal cluster CV (%), mean (SD)	Avg. carb intake (g), mean (SD)	PPGRs in outlier cluster	Peak BG (mmol/L), mean (SD)	Internal cluster CV (%), mean (SD)	Avg. carb intake (g), mean (SD)	*F* test *(df)*	*P* value
522	Breakfast	3	12.3 (15.3)	9.1 (2.4)	33.0 (2.5)	49.1 (25.7)	N/A^d^	N/A	N/A	N/A	0.64 (2, 23)	.53
	Lunch	2	32.0 (39.5)	11.9 (4.9)	23.3 (17.8)	56.8 (33.3)	1	9.4 (5.3)	56.7 (0.8)	15 (0.0)	1.5 (1, 32)	.22
	Dinner	2	27.5 (24.7)	7.8 (2.7)	28.5 (3.7)	47.3 (2.6)	4	8.3 (3.8)	45.9 (0.3)	—^e^	0.09 (1, 30)	.07
540	Breakfast	3	16.7 (24.5)	11.0 (4.0)	28.8 (9.0)	38.2 (33.9)	5	7.7 (3.7)	48.6 (0.2)	55.0 (49.5)	1.62 (1, 30)	.21
	Lunch	3	23.7 (26.3)	9.7 (2.3)	19.7 (4.8)	60.6 (2.8)	4	8.0 (3.6)	45 (0.3)	100.0 (0.0)	0.66 (3, 29)	.58
	Dinner	4	16.5 (23.1)	9.6 (108)	31.4 (3.9)	62.2 (15.6)	10	8.0 (3.8)	46.9 (0.2)	20.5 (27.6)	1.18 (4, 22)	.34
544	Breakfast	1	51.0 (0.0)	9.7 (2.5)	25.4 (0.1)	41.1 (17.2)	N/A	N/A	N/A	N/A	Not performed due to one cluster formed	N/A
	Lunch	2	27.5 (27.5)	12.3 (3.1)	29.4 (0.6)	102.6 (4.9)	N/A	N/A	N/A	N/A	0.61 (1, 52)	.44
	Dinner	1	75.0 (0.0)	9.8 (3.1)	31.8 (0.1)	68.2 (38.2)	3	9.5 (5.0)	52.9 (0.4)	93.9 (64.3)	1.19 (1, 75)	.28
559	Breakfast	2	28.5 (34.6)	8.5 (4.2)	28.7 (8.6)	31.3 (3.6)	1	8.0 (0.0)	52.6 (0.6)	30.0 (0.0)	0.56 (2, 55)	.57
	Lunch	2	29.5 (2.1)	9.4 (3.3)	29.4 (1.6)	34.4 (3.2)	3	9.9 (4.9)	50 (0.4)	3.0 (2.9)	4.66 (2, 59)	.01
	Dinner	2	26.0 (19.7)	10.4 (4.2)	28.4 (1.4)	42.0 (5.8)	2	9.1 (5.0)	54.6 (0.5)	32.5 (17.6)	1.08 (2, 51)	.35
563	Breakfast	6	10.2 (16.7)	8.5 (1.7)	33.4 (3.3)	24.2 (9.7)	4	11.1 (3.5)	546 (0.4)	36.0 (0.8)	0.71 (6, 47)	.64
	Lunch	1	59.0 (0.0)	9.1 (2.3)	25.8 (0.04)	29.9 (14.5)	2	7.0 (4.0)	52.9 (0.4)	40.0 (0.0)	0.48 (1, 47)	.49
	Dinner	2	37.0 (27.0)	9.1 (2.4)	26.5 (0.5)	38.9 (10.3)	4	8.6 (4.5)	51.9 (0.3)	20.0 (21.2)	3.48 (2, 43)	.04
567	Breakfast	1	49.0 (0.0)	9.3 (2.7)	28.8 (0.1)	72.6 (16.0)	2	6.5 (2.8)	51.3 (0.3)	—	Not performed due to one cluster formed	N/A
	Lunch	3	20.7 (20.8)	8.3 (1.7)	31.1 (3.5)	81.2 (9.5)	5	9.5 (6.2)	65.4 (0.4)	87.0 (0.0)	1.48 (2, 8)	.28
	Dinner	2	35.0 (46.7)	12.3 (2.7)	33.2 (2.5)	83.2 (0.0)	2	9.7 (5.1)	52.8 (0.5)	110.0 (0.0)	0.73 (1, 5)	.43
570	Breakfast	1	51.0 (0.0)	13.0 (3.5)	26.6 (0.1)	101.4 (39.1)	N/A	N/A	N/A	N/A	Not performed due to one cluster formed	N/A
	Lunch	3	17.0 (23.4)	7.4 (2.9)	29.4 (5.8)	125.2 (16.0)	N/A	N/A	N/A	N/A	0.84 (2, 46)	.44
	Dinner	3	23.0 (34.6)	9.4 (5.1)	24.0 (9.8)	113.7 (27.3)	1	5.5 (2.2)	39.4 (0.3)	125 (0.0)	0.94 (3, 65)	.43
575	Breakfast	1	57.0 (0.0)	4.6 (0.2)	4.4 (0.0)	40.8 (13.9)	N/A	N/A	N/A	N/A	Not performed due to one cluster formed	N/A
	Lunch	1	90.0 (0.0)	4.6 (0.2)	3.8 (0.0)	38.9 (23.1)	N/A	N/A	N/A	N/A	Not performed due to one cluster formed	N/A
	Dinner	1	108.0 (0.0)	4.6 (0.2)	4.0 (0.0)	44.9 (27.2)	N/A	N/A	N/A	N/A	Not performed due to one cluster formed	N/A
584	Breakfast	3	17.6 (22.9)	11.6 (5.7)	27.4 (2.9)	59.3 (1.2)	N/A	N/A	N/A	N/A	0.59 (2, 32)	.56
	Lunch	2	24.0 (31.1)	7.3 (3.1)	23.4 (12.8)	57.6 (3.4)	N/A	N/A	N/A	N/A	0.56 (1, 27)	.46
	Dinner	2	41.0 (48.1)	12.1 (2.7)	30.2 (4.4)	55.9 (5.8)	2	7.1 (3.8)	54.3 (0.5)	60.0 (0.0)	0.34 (2, 50)	.71
588	Breakfast	1	55.0 (0.0)	9.8 (2.4)	14.9 (4.0)	14.8 (3.9)	N/A	N/A	N/A	N/A	Not performed due to one cluster formed	N/A
	Lunch	2	37 (46.7)	12.3 (5.0)	28.9 (1.0)	37.9 (0.6)	2	16.2 (7.1)	43.8 (0.7)	39.0 (17.0)	0.01 (2, 73)	.99
	Dinner	3	39.3 (21.9)	9.7 (2.1)	25.8 (5.1)	37.0 (1.1)	4	8.9 (4.1)	46.2 (0.3)	31.3 (14.4)	0.04 (3, 114)	.99
591	Breakfast	1	66.0 (0.0)	7.5 (2.6)	34.5 (0.1)	28.5 (11.4)	7	8.0 (4.8)	60.4 (0.3)	30.1 (16.3)	12 (1, 71)	.73
	Lunch	2	34.5 (30.4)	7.4 (2.7)	33.2 (3.1)	32.6 (7.1)	3	8.9 (4.8)	53.3 (0.4)	32.3 (8.1)	2.39 (2, 68)	.099
	Dinner	2	43.5 (44.5)	7.4 (2.9)	28.7 (0.4)	30.3 (1.8)	5	7.0 (4.0)	56.4 (0.3)	45 (14.5)	1.92 (2, 81)	.15
596	Breakfast	1	64.0 (0.0)	8.9 (3.0)	33.6 (0.1)	25.4 (6.7)	N/A	N/A	N/A	N/A	Not performed due to one cluster formed	N/A
	Lunch	1	7.0 (0.0)	7.9 (2.8)	35.4 (0.0)	24.4 (15.0)	3	8.1 (4.4)	53.6 (0.4)	18 (15.7)	0.53 (1, 106)	.47
	Dinner	2	50.0 (66.5)	6.5 (2.7)	30.1 (3.2)	26.6 (0.8)	N/A	N/A	N/A	N/A	0.68 (2, 92)	.51

a The ANOVA results (*F* value [degrees of freedom], *P* value) compare the effect of the average carbohydrate intake and the cluster formation.

b PPGRs: postprandial glycemic responses.

c BG: blood glucose.

d N/A: not applicable.

e —: average carbs not known.

## Discussion

### Principal Findings and Comparison With Previous Works

In this study, data from 12 people with T1D were analyzed. Each participant contributed 9900 BG measurements, which were used to generate and cluster over 200 isolated PPGRs per person. CV-based clustering identified stable, participant-specific PPGR patterns, averaging 2.4 for breakfast, 2.7 for lunch, and 3.1 for dinner with most cluster errors below the CGM’s intrinsic error. Carbohydrate intake was largely consistent within clusters, with significant variation in only 2 of 36 meal-participant combinations. The large effect size (Cohen *d*=1.20) underscores the robustness of this approach. These findings demonstrate the intraindividual reproducibility of the PPGRs within a meal category under free-living conditions in people with T1D, highlighting the possibility to predict the PPGR to a given meal in an individual, a concept already validated in healthy individuals [15] and people living with type 2 diabetes [5]. Using PPGR clusters to highlight how an individual responds to a specific meal type provides insight into the overall meal metabolism without requiring onerous additional information from the people with T1D. This could outline appropriate insulin doses for each PPGR cluster that would optimize TIR. Enhancing the PPGR clustering framework presented here without increasing the burden of disease management, through the utilization of meal tags associated with each PPGR event, could ease the burden of nutrition reporting.

Using the CV as a clustering metric for PPGRs is clinically advantageous because a PPGR-specific CV would be expected to be significantly higher than a 24-hour CV, meaning that applying the 24-hour clinical threshold of 36% ensures tighter internal cohesion than the recommended glycemic variability limit. In a comparative analysis, k-means clustering with dynamic time warping produced moderate cohesion, with 50% (6/12) of participants forming clusters across at least 1 time-of-day period when set to 3 clusters (59% of these meeting the CV threshold) and 58% (7/12) when set to 4 clusters (61% meeting the threshold). By contrast, our proposed method achieves markedly superior performance: in all clusters containing more than 1 PPGR, 100% meet the clinical CV threshold, and cluster formation covers all meal categories for every participant, ensuring both clinical relevance and complete coverage.

In the dataset observed within this work, carbohydrate intake alone does not typically predict which cluster a corresponding PPGR would align to, highlighting the importance of overall meal composition and the physiological factors of the individual in determining the PPGR outcome. In those meals where the average PPGR clusters do not meet the target CV, there is a large discrepancy in the number of PPGRs isolated to each cluster, with many of the clusters resulting from outliers that skew the average CV (as seen in Table S1 in Multimedia Appendix 1). Outliers are to be expected given the real-world setting of the dataset.

The limitations of using carbohydrate alone to predict the PPGR are evident and reflect the known limitations of carbohydrate counting, with studies showing a 20% mean error in carbohydrate estimates in people with T1D [53]. The complexity of carbohydrate-based insulin dosing is compounded by the varying rates of glucose absorption from different types of carbohydrates or meals with multiple components, making them nutritionally complex [53,54]. The absorption of digestible carbohydrates in comparison to simple glucose, known as the glycemic index (GI), is well researched [4,20,54]; however, when used in the real world, with complex mixed meals, the prediction of the PPGR using solely GI has shown low precision [13,55]. The unpredictable interaction of several factors, dietary and otherwise [56-58], contributes to the complexity and interpersonal nature of the management of the PPGR [59].

The number of clusters formed per meal type varies with time of day, highlighting the significant impact of meal timing on the PPGR. The PPGR response to meals with the same nutritional breakdown, consumed at different times of day, is expected to be greater during the night versus during the day [60]. Current treatment guidelines considering insulin-to-carbohydrate ratios, which fluctuate throughout the day, support the argument that carbohydrate content alone should not dictate the PPGR [61,62]. This enhances the argument reflected in this work that carbohydrate content of a meal alone should not be the only determining factor of the PPGR [59,63]. The work here suggests that the PPGR is decided by a combination of the complete nutritional intake and the individual’s physiology, which is supported by the intraindividual PPGR reproducibility to identical meals shown in 800 people [15]. Overall, this highlights the need for the development of a personalized prediction model based on unique PPGR events [58].

Meal timing, along with nutritional composition, has a marked effect on the PPGR [17,24,63], resulting in meals of identical nutritional profiles eliciting different PPGRs when consumed at different times of day (ie, breakfast vs dinner). The treatment relevance of the reproducibility of a PPGR to similar meals at a similar time of day is exacerbated by the fact that habit is a proven driver of food consumption [64,65], both in the type of food and in portion size [66]. To enhance this identified habitual human nature, people with T1D are encouraged to “establish a routine” in the early stages after diagnosis [40]. These factors that enhance the repetitive nature of PPGRs offer the possibility to streamline treatment plans through clustering the PPGRs based on pattern recognition. The traditional treatment strategies of carbohydrate-based insulin dosing have limitations, especially for the case of high fat, high protein foods, which are consistently problematic to manage, along with breakfast cereals possibly due to the high fiber and sugar content [67]. It has been indicated that a person with T1D will use previous experience as a determinant for the bolus dose needed to cover a meal [68]. The optimal indicators of the PPGR in a person with T1D are the carbohydrate and fat content of a meal [20,69]. However, reporting multiple macronutrients compounds the burden on people with T1D, increasing the likelihood of reporting errors. Due to the complexity of PPGR prediction, along with the limitations of carbohydrate counting as a predictor alone, it has been reported that the bolus insulin dose needed to cover the meal consumed was inaccurately estimated in 64% of cases [70]. Therefore, assigning PPGRs to clusters based on an individual’s unique response to a meal would simplify treatment planning and reduce the reliance on determining meal carbohydrate content. The presented PPGR-focused clustering algorithm offers an alternative to traditional evaluation methods, such as area under the curve based on GI, which fail to capture individual physiological responses to meals [59].

The clear establishment of PPGR clusters reflects the recurrent behaviors of people with T1D, potentially offering clinical insights for insulin dose decisions and treatment adjustments to optimize TIR. Utilizing PPGR clusters to predict BG values from the PPGR of a previously consumed meal could offer a reduction in patient anxiety and diabetes burden, due to the foreknowledge of upcoming glycemic fluctuations, ultimately easing the management of T1D. Although this work investigates real-world scenarios, the outcomes are supported by the discernible correlation between PPGRs shown to standardized meals [71]. The assessment of CGM data for patterns shows that interday similarities tend to be higher in older people with T1D [18], which reflects the population in the Ohio dataset used here, whose average age is in the range of 30.9‐56.4 years. The presence of repeatable PPGRs in the real world, as seen in this work, may be attributed to factors such as insulin utilization and the habitual nature of dietary intake and lifestyles. This habitual nature of people’s eating patterns, along with the encouraged ritualistic nature of disease management in T1D, will allow the use of PPGR clusters to enhance a feedback loop to better predict BG levels. The work presented here provides an initial framework for prediction that is independent of the initial BG level and not solely reliant on identifying the amount of carbohydrates consumed, suggesting a more physiological-based glycemic response to food. A bolus targeting solution algorithm based on the PPGR-focused clustering algorithm presented here would allow for an insulin adjustment plan to optimize time in range for each isolated PPGR cluster.

### Limitations and Future Work

This study presents a PPGR clustering method that incorporates the full 4-hour BG profile, enabling reduced glycemic variability and contextual insights into individual responses to specific meal types. While promising, limitations include reliance on self-reported meals including carbohydrate intake, which is prone to consistent under- or overreporting within individuals [72], and omission of insulin dose, insulin-on-board, and carbohydrates-on-board, all of which influence PPGR. The dataset was derived from multiple clinical studies with nonstandardized insulin regimens, and insulin sensitivity was treated as an independent variable. Future work will address these factors by incorporating dose, timing, and administration data to improve modeling accuracy.

Even with reproducible PPGR clusters, translating findings into insulin dosing strategies remains complex due to the highly individualized nature of bolus timing and dose optimization [3,73]. In both research datasets (eg, OhioT1DM) and real-world contexts, inconsistent meal logging presents challenges. To mitigate this, we propose a meal-tagging mechanism to link PPGRs via pattern recognition rather than precise carbohydrate counts (Lubasinski et al, unpublished data, 2025) [68], enabling clusters to form based on similar nutritional profiles. This will reduce reporting burden, align with natural logging habits, and provide proactive predictions for upcoming meals, including tailored bolus recommendations aimed at optimizing TIR while lowering cognitive load.

Limitations also include a small sample size, reducing statistical power and generalizability. This work is exploratory and should be interpreted with caution due to potential sampling bias. Broader validation is essential, and our methodology has already been applied to a larger public dataset [74] and replicated in follow-up work (Lubasinski et al, unpublished data, 2025). Future multicenter studies with more diverse populations are recommended to strengthen statistical power, enable subgroup analysis, and support the development of scalable, individualized glycemic prediction and adaptive insulin dosing models.

### Conclusions

The clinically relevant and safe PPGR clustering technique developed in this study categorizes PPGRs into distinct PPGR clusters based on the participants’ unique response to a meal. This offers a framework for a more personalized and adaptable way to manage PPGRs, independent of initial BG levels or carbohydrate consumption. The reproducibility of PPGRs offers clinical significance through insights for insulin dosing decisions and treatment adjustments to optimize TIR, which may indicate priming insulin doses to flatten the PPGR spike. This will alleviate patient anxiety by providing advance knowledge of glycemic fluctuations and by learning from the past behaviors and outcomes within each cluster. Notably, this work challenges the conventional notion that carbohydrate intake alone determines PPGRs. In summary, we found that PPGRs are reproducible, resulting from a complex interplay of an individual’s unique physiology and their overall nutritional intake, underscoring the need for personalized prediction models.

## Supplementary material

10.2196/68821 Multimedia Appendix 1 Breakdown of each of the clusters formed within each meal category per participant.

## Abbreviations

BG: blood glucose
CGM: continuous glucose monitor
CV: coefficient of variability
GI: glycemic index
PPGR: postprandial glycemic response
PwT1D: people with T1D
T1D: type 1 diabetes
TIR: time in range
