# Supplementary material for: Coefficient of Variation to Assess the Reproducibility of Meal-Induced Glycemic Responses: Development of a Clustering Algorithm
Source: JMIR Diabetes. 2025 Nov 20;10:e68821. doi: 10.2196/68821 (PMC12633838; doi:10.2196/68821)
Supplement: Multimedia Appendix 1 [file diabetes-v10-e68821-s001.docx]

Supplementary Table 1: Breakdown of each of the clusters formed within each meal category per participant.

| Participant | MealCategory | Cluster Number | No of PPGRs | Mean_Peak | Std Deviation | Std Error | CV (%) | Carbs_g | ANOVA |
| --- | --- | --- | --- | --- | --- | --- | --- | --- | --- |
| 522 | Breakfast | 1 | 30 | 6.7 | 2.0 | 0.1 | 30.5 | 58.5 | F (2,23) = 0.64  *p* = .535 |
|  | Breakfast | 2 | 5 | 9.0 | 3.0 | 0.2 | 33.0 | 68.8 |  |
|  | Breakfast | 3 | 2 | 11.5 | 4.1 | 0.5 | 35.5 | 20.0 |  |
|  | Lunch | 1 | 60 | 8.5 | 3.0 | 0.1 | 35.9 | 56.8 | F (1,32) = 1.53  *p* = .225 |
|  | Lunch | Outliers | 1 | 9.4 | 5.3 | 0.8 | 56.7 | 15.0 |  |
|  | Lunch | 3 | 4 | 15.4 | 1.6 | 0.1 | 10.7 | - |  |
|  | Dinner | 1 | 45 | 9.7 | 3.0 | 0.1 | 31.1 | 45.4 | F (1,30) = 0.09  *p* = .764 |
|  | Dinner | 2 | 10 | 5.8 | 1.5 | 0.1 | 25.8 | 49.2 |  |
|  | Dinner | Outliers | 4 | 8.3 | 3.8 | 0.3 | 45.9 |  |  |
| 540 | Breakfast | 1 | 45 | 7.3 | 2.4 | 0.1 | 33.2 | 46.1 | F (3,30) = 1.62  *p* = .205 |
|  | Breakfast | 2 | 3 | 15.1 | 2.8 | 0.3 | 18.5 | 67.5 |  |
|  | Breakfast | 6 | 2 | 10.5 | 3.6 | 0.4 | 34.9 | 1.0 |  |
|  | Breakfast | Outliers | 5 | 7.7 | 3.7 | 0.2 | 48.6 | 55.0 |  |
|  | Lunch | 1 | 54 | 7.3 | 2.3 | 0.0 | 31.0 | 58.9 | F (3,29) = 0.66  *p* = .580 |
|  | Lunch | 2 | 9 | 11.7 | 2.9 | 0.1 | 24.4 | 63.8 |  |
|  | Lunch | 3 | 8 | 10.2 | 3.4 | 0.2 | 33.7 | 59.0 |  |
|  | Lunch | Outliers | 4 | 8.0 | 3.6 | 0.3 | 45.0 | 100 |  |
|  | Dinner | 1 | 51 | 6.8 | 2.3 | 0.0 | 34.1 | 64.3 | F (4,22) = 1.18  *p* = .343 |
|  | Dinner | 3 | 8 | 11.0 | 3.1 | 0.2 | 28.3 | 51.0 |  |
|  | Dinner | 5 | 5 | 12.8 | 3.5 | 0.3 | 27.7 | 83.5 |  |
|  | Dinner | 9 | 2 | 7.7 | 2.7 | 0.3 | 35.4 | 50.0 |  |
|  | Dinner | Outliers | 10 | 8 | 3.8 | 0.2 | 46.9 | 20.5 |  |
| 544 | Breakfast | 1 | 51 | 9.7 | 2.5 | 0.1 | 25.4 | 41.1 | Not performed due to one Cluster formed |
|  | Lunch | 1 | 47 | 10.1 | 3.0 | 0.1 | 29.8 | 99.2 | F (1,52) = 0.61  *p* = .439 |
|  | Lunch | 2 | 8 | 14.5 | 4.2 | 0.2 | 29.0 | 106.1 |  |
|  | Dinner | 1 | 75 | 9.8 | 3.1 | 0.1 | 31.8 | 68.2 | F (1,75) = 1.19  *p* = .279 |
|  | Dinner | Outliers | 3 | 9.5 | 5.0 | 0.4 | 52.9 | 93.9 |  |
| 559 | Breakfast | 1 | 53 | 11.5 | 4.0 | 0.1 | 34.8 | 33.9 | F (2,55) = 0.56  *p* = .573 |
|  | Breakfast | 2 | 4 | 5.5 | 1.2 | 0.1 | 22.6 | 28.8 |  |
|  | Breakfast | Outliers | 1 | 8.0 | 4.2 | 0.6 | 52.6 | 30.0 |  |
|  | Lunch | 1 | 31 | 7.0 | 2.0 | 0.1 | 28.3 | 32.2 | F (2,59) = 4.66  *p* = .013 |
|  | Lunch | 2 | 28 | 11.7 | 3.6 | 0.1 | 30.5 | 36.7 |  |
|  | Lunch | Outliers | 3 | 9.9 | 4.9 | 0.7 | 50.0 | 63.3 |  |
|  | Dinner | 1 | 40 | 7.4 | 2.2 | 0.1 | 29.4 | 37.9 | F (2,51) = 1.08  *p* = .348 |
|  | Dinner | 2 | 12 | 13.4 | 3.7 | 0.2 | 27.4 | 46.2 |  |
|  | Dinner | Outliers | 2 | 9.1 | 4.96 | 0.5 | 54.6 | 32.5 |  |
| 563 | Breakfast | 1 | 44 | 8.7 | 2.4 | 0.1 | 27.1 | 26.0 | F (6,47) = 0.71  *p* = .637 |
|  | Breakfast | 2 | 3 | 9.4 | 3.3 | 0.3 | 35.2 | 15.5 |  |
|  | Breakfast | 3 | 8 | 5.6 | 1.9 | 0.1 | 33.3 | 25.9 |  |
|  | Breakfast | 4 | 2 | 7.6 | 2.7 | 0.3 | 35.7 | 12.5 |  |
|  | Breakfast | 6 | 2 | 10.6 | 3.5 | 0.4 | 33.5 | 40.0 |  |
|  | Breakfast | 7 | 2 | 9.0 | 3.2 | 0.3 | 35.8 | 25.5 |  |
|  | Breakfast | Outliers | 4 | 11.1 | 3.5 | 0.5 | 36.5 | 36.0 |  |
|  | Lunch | 1 | 59 | 9.1 | 2.3 | 0.0 | 25.8 | 29.9 | F (1,47) = 0.48  *p* = .493 |
|  | Lunch | Outliers | 2 | 7.0 | 3.7 | 4.0 | 52.9 | 40.0 |  |
|  | Dinner | 1 | 54 | 7.4 | 2.0 | 0.0 | 26.8 | 31.7 | F (2,43) = 3.48  *p* = .040 |
|  | Dinner | 2 | 20 | 10.8 | 2.8 | 0.1 | 26.1 | 46.2 |  |
|  | Dinner | Outliers | 4 | 8.6 | 4.5 | 0.3 | 51.9 | 20 |  |
| 567 | Breakfast | 1 | 49 | 9.3 | 2.7 | 0.1 | 28.8 | 72.6 | Not performed due to one Cluster formed |
|  | Breakfast | Outliers | 2 | 5.5 | 2.8 | 0.3 | 51.3 |  |  |
|  | Lunch | 1 | 44 | 10.2 | 2.9 | 0.1 | 28.1 | 67.3 | F (2,8) = 1.48  *p* = .285 |
|  | Lunch | 2 | 4 | 7.9 | 2.4 | 0.2 | 30.2 | 95.0 |  |
|  | Lunch | 3 | 14 | 6.8 | 2.4 | 0.1 | 35.0 | - |  |
|  | Lunch | Outliers | 5 | 9.5 | 6.2 | 0.4 | 65.4 | 87 |  |
|  | Dinner | 1 | 68 | 10.4 | 3.3 | 0.1 | 31.4 | 83.2 | F (1,5) = 0.73  *p* = .432 |
|  | Dinner | 2 | 2 | 14.2 | 5.0 | 0.5 | 34.9 | - |  |
|  | Dinner | Outliers | 2 | 9.7 | 5.1 | 0.5 | 52.8 | 110 |  |
| 570 | Breakfast | 1 | 51 | 13.0 | 3.5 | 0.1 | 26.6 | 101.4 | Not performed due to one Cluster formed |
|  | Lunch | 1 | 44 | 10.6 | 2.9 | 0.1 | 27.8 | 111.1 | F (2,46) = 0.84  *p* = .439 |
|  | Lunch | 2 | 2 | 6.5 | 2.3 | 0.2 | 35.8 | 142.5 |  |
|  | Lunch | 3 | 5 | 5.0 | 1.2 | 0.1 | 24.5 | 122.0 |  |
|  | Dinner | 1 | 63 | 8.6 | 3.0 | 0.1 | 35.0 | 101.1 | F (3,65) = 0.94  *p* = .428 |
|  | Dinner | 2 | 3 | 14.8 | 2.4 | 0.2 | 16.2 | 95.0 |  |
|  | Dinner | 3 | 3 | 4.7 | 1.0 | 0.1 | 20.8 | 145.0 |  |
|  | Dinner | Outliers | 1 | 5.5 | 2.2 | 0.3 | 39.4 | 125.0 |  |
| 575 | Breakfast | 1 | 57 | 4.6 | 0.2 | 0.0 | 4.4 | 40.8 | Not performed due to one Cluster formed |
|  | Lunch | 1 | 90 | 4.6 | 0.2 | 0.0 | 3.8 | 38.9 | Not performed due to one Cluster formed |
|  | Dinner | 1 | 108 | 4.6 | 0.2 | 0.0 | 4.0 | 44.9 | Not performed due to one Cluster formed |
| 584 | Breakfast | 1 | 44 | 10.4 | 3.2 | 0.1 | 30.6 | 57.9 | F (2,32) = 0.59  *p*= .561 |
|  | Breakfast | 2 | 2 | 17.8 | 4.5 | 0.5 | 25.2 | 60.0 |  |
|  | Breakfast | 3 | 7 | 6.5 | 1.7 | 0.1 | 26.3 | 60.0 |  |
|  | Lunch | 1 | 46 | 9.5 | 3.1 | 0.1 | 32.5 | 55.2 | F (1,27) = 0.56  *p* = .460 |
|  | Lunch | 2 | 2 | 5.1 | 0.7 | 0.1 | 14.3 | 60.0 |  |
|  | Dinner | 1 | 75 | 10.2 | 2.8 | 0.0 | 27.1 | 51.8 | F (2,50) = 0.34  *p* = .712 |
|  | Dinner | 2 | 7 | 14.0 | 4.7 | 0.3 | 33.3 | 60.0 |  |
|  | Dinner | Outliers | 2 | 7.1 | 3.8 | 0.5 | 54.3 | 60.0 |  |
| 588 | Breakfast | 1 | 55 | 9.8 | 2.4 | 0.0 | 24.3 | 14.9 | Not performed due to one Cluster formed |
|  | Lunch | 1 | 70 | 8.8 | 2.6 | 0.0 | 29.6 | 38.3 | F (2,73) = 0.01  *p* = .992 |
|  | Lunch | 3 | 4 | 15.8 | 4.5 | 0.3 | 28.3 | 37.5 |  |
|  | Lunch | Outliers | 2 | 16.2 | 7.1 | 0.7 | 43.8 | 39.0 |  |
|  | Dinner | 1 | 64 | 7.4 | 2.0 | 0.0 | 26.8 | 38.0 | F (3,117) = 0.04  *p* = .998 |
|  | Dinner | 2 | 32 | 10.4 | 2.1 | 0.1 | 20.3 | 37.1 |  |
|  | Dinner | 3 | 22 | 11.5 | 3.5 | 0.1 | 30.2 | 35.8 |  |
|  | Dinner | Outliers | 4 | 8.9 | 4.1 | 0.3 | 46.2 | 31.3 |  |
| 591 | Breakfast | 1 | 66 | 7.5 | 2.6 | 0.0 | 34.5 | 28.5 | F (1,71) = 0.12  *p* = .732 |
|  | Breakfast | Outliers | 7 | 8.0 | 4.8 | 0.3 | 60.4 | 30.1 |  |
|  | Lunch | 1 | 56 | 9.3 | 3.3 | 0.1 | 35.4 | 37.6 | F (2,68) = 2.39  *p* = .099 |
|  | Lunch | 3 | 13 | 5.6 | 1.7 | 0.1 | 31.0 | 27.6 |  |
|  | Lunch | Outliers | 3 | 8.9 | 4.8 | 0.4 | 53.3 | 32.3 |  |
|  | Dinner | 1 | 75 | 9.5 | 2.7 | 0.0 | 29.0 | 31.6 | F (2,81) = 1.92  *p* = .153 |
|  | Dinner | 3 | 12 | 5.4 | 1.5 | 0.1 | 28.4 | 29.0 |  |
|  | Dinner | Outliers | 5 | 7.03 | 3.96 | 0.3 | 56.4 | 45 |  |
| 596 | Breakfast | 1 | 64 | 8.9 | 3.0 | 0.1 | 33.6 | 25.4 | Not performed due to one Cluster formed |
|  | Lunch | 1 | 107 | 7.9 | 2.8 | 0.0 | 35.4 | 24.4 | F (1,106) = 0.53  *p* = .468 |
|  | Lunch | Outliers | 3 | 8.1 | 4.4 | 0.4 | 53.6 | 18.0 |  |
|  | Dinner | 1 | 97 | 8.4 | 2.7 | 0.0 | 32.4 | 27.1 | F (2,92) = 0.68  *p* = .510 |
|  | Dinner | 2 | 3 | 4.6 | 1.3 | 0.1 | 27.8 | 26.0 |  |
|  | Dinner | Outliers | 1 | 5.2 | 1.9 | 0.3 | 37.1 | 9.0 |  |
